# Efficacy and Safety of Distal Radial Access for Transcatheter Arterial Chemoembolization (TACE) of the Liver

**DOI:** 10.3390/jpm13040640

**Published:** 2023-04-07

**Authors:** Roberto Minici, Raffaele Serra, Marco Giurdanella, Marisa Talarico, Maria Anna Siciliano, Gianpaolo Carrafiello, Domenico Laganà

**Affiliations:** 1 Radiology Unit, Dulbecco University Hospital, 88100 Catanzaro, Italy; miniciroberto@gmail.com (R.M.); marco-giurdanella@virgilio.it (M.G.); 2 Vascular Surgery Unit, Department of Medical and Surgical Sciences, Magna Graecia University of Catanzaro, Dulbecco University Hospital, 88100 Catanzaro, Italy; rserra@unicz.it; 3 Cardiology Unit, Dulbecco University Hospital, 88100 Catanzaro, Italy; marisa.talarico@icloud.com; 4 Oncology Unit, Dulbecco University Hospital, 88100 Catanzaro, Italy; maria.anna.siciliano@hotmail.com; 5 Department of Health Sciences, Università Degli Studi di Milano, 20122 Milan, Italy; gianpaolo.carrafiello@unimi.it; 6 Department of Experimental and Clinical Medicine, Magna Graecia University of Catanzaro, 88100 Catanzaro, Italy

**Keywords:** distal radial artery, distal radial access, dRA, anatomical snuff box, TACE, liver chemoembolization, DEB-TACE, radial access, femoral access, vascular access

## Abstract

Background: The distal radial artery has emerged as an alternative vascular-access site to conventional transfemoral and transradial approaches. The main advantage over the conventional transradial route is the reduced risk of radial artery occlusion, especially in those patients who, for various clinical reasons, have to undergo repeated endovascular procedures. This study aims to assess the efficacy and safety of distal radial access for transcatheter arterial chemoembolization of the liver. Methods: This investigation is a single-center retrospective analysis of 42 consecutive patients who had undergone, from January 2018 to December 2022, transcatheter arterial chemoembolization of the liver with distal radial access for intermediate-stage hepatocellular carcinoma. Outcome data were compared with a retrospectively constituted control group of 40 patients undergoing drug-eluting beads-transcatheter arterial chemoembolization with femoral access. Results: Technical success was achieved in all cases, with a 2.4% conversion rate for distal radial access. A superselective chemoembolization was performed in 35 (83.3%) cases of distal radial access. No episode of radial artery spasm or radial artery occlusion occurred. No significant differences in efficacy and safety were observed between the distal radial access group and the femoral access group. Conclusions: Distal radial access is effective, safe, and comparable to femoral access in patients undergoing transcatheter arterial chemoembolization of the liver.

## 1. Introduction

In recent years, the use of transradial access (TRA) in endovascular procedures has highlighted many advantages over the transfemoral access route, including the following: fast recovery, fewer severe hemorrhagic events, less vascular complications, less adverse cardiovascular events and mortality, ease of hemostasis, and patient preference [1,2,3,4,5]. More recently, the distal radial artery (dRA) has emerged as an alternative vascular access site to conventional transfemoral and transradial approaches, with the reduced risk of radial artery occlusion (RAO) described as the main additional benefit over the conventional transradial access [6]. The aforementioned advantage is of particular value in those patients who, for various clinical reasons, have to undergo repeated endovascular procedures (TACEs, PCIs, etc.). 

While an increasing number of investigations have shown that distal radial artery access has a comparable safety and efficacy profile to the conventional transradial approach for interventional cardiology procedures [7], few case-series studies have investigated this approach for interventional radiology procedures with no data collected exclusively for chemoembolization of the liver [8,9,10].

This study aims to assess the efficacy and safety of distal radial access for transcatheter arterial chemoembolization (TACE) of the liver, attempting to cover a current lack of data in the literature.

## 2. Materials and Methods

### 2.1. Study Design

This study is a single-center analysis of prospectively collected data of consecutive patients who had undergone, from January 2018 to December 2022, a drug-eluting beads-transcatheter arterial chemoembolization (DEB-TACE) of the liver with distal radial access. Inclusion criteria were (I) intermediate-stage (B) hepatocellular carcinoma (HCC) according to the Barcelona Clinic Liver Cancer (BCLC) staging system [11,12,13], not previously treated with TACE, and diagnosed with histological assessment or non-invasive imaging-based criteria according to the European Association for the Study of the Liver [14]; (II) Child–Pugh stage A or B and Eastern Cooperative Oncology Group performance status [15] grade 0; (III) age between 18 and 75 years; (IV) no previous endovascular treatments or vascular accesses on arteries of the same upper limb of the distal radial access; (V) distal radial access as vascular access site and patency of the radiopalmar arch assessed by the Barbeau test [16]; (VI) evaluation by a multidisciplinary team of hepatologist, oncologist, liver surgeon, and interventional radiologist. The exclusion criteria were: (I) nonpalpable radial artery at the wrist; (II) serum creatinine levels >2.0 mg/dL; (III) platelet count <50,000/μL and/or international normalized ratio >1.5; (IV) serum bilirubin level ≥3 mg/dL; (V) chemotherapeutic agent administration contraindications; (VI) distal radial artery diameter less than 2 mm.

Patients undergoing DEB-TACE with a transfemoral approach during the same study interval were retrospectively evaluated to constitute a control group. The same indications and treatment technique as in the distal radial access group were applied to the control group, except for access-site management. The choice of the vascular access site was determined by the multidisciplinary team after a comprehensive evaluation, also taking into account the preferences expressed by the patient and the vascular accesses available.

### 2.2. Treatment

Within 3 weeks prior to each treatment, each patient underwent a clinical, laboratory, and imaging evaluation. Imaging evaluation was performed by contrast-enhanced computed tomography (CT) and/or gadolinium-enhanced magnetic resonance imaging (MRI) with a multiphase liver-imaging protocol. The laboratory evaluation also included inflammation-based scores (e.g., neutrophil-to-lymphocyte ratio) in consideration of their growing impact on the prognosis highlighted in numerous recent studies [17]. In the case of antiplatelet therapy, clopidogrel was discontinued 5 days before treatment, while acetylsalicylic acid was continued. The endovascular procedure was performed in dedicated angiographic suites. All patients were given a proton-pump inhibitor (Omeprazole 40 mg i.v.), an antiemetic drug (Metoclopramide 10 mg i.v.), and an analgesic drug (Ketorolac-Tromethamine 20 mg i.v.), at the start of the procedure. Preoperative ultrasound (US)-guided measurement of distal radial artery diameter was performed using the anatomical snuffbox as the measurement site. The patency of the distal radial artery at the snuffbox was evaluated by doppler ultrasound, and a Barbeau test was performed in each patient. The patient’s forearm was placed in a neutral semi-pronated position with a small towel or gauze roll, which was done to improve patient comfort and to pull the thumb down, making the radial fossa more prominent. Skin disinfection of the vascular access site was made with a povidone–iodine solution. After local anesthesia with subcutaneous injection of lidocaine (2 mL of lidocaine 2%), US-guided arterial puncture of the distal radial artery was performed with the 21-gauge micro-puncture needle of the Glidesheath Slender™ Introducer Kit (Terumo Corp, Tokyo, Japan). After successful 0.018” wire insertion under fluoroscopic guidance, the needle was removed, and a hydrophilic 5Fr introducer sheath (Glidesheath Slender™; Terumo Corp, Tokyo, Japan) was positioned. A spasmolytic cocktail was then administered (200 mcg of Nitroglycerin and 2.5 mg of Verapamil), as in Azizi et al. [18], to prevent radial artery spasm. The intra-arterial administration of unfractionated heparin (2500 IU) to reduce the incidence of radial artery occlusion (RAO) was inconstant due to the variable bleeding risk in patients with hepatopathy; in patients with aPTT prolongation or low platelet count (less than 100,000 platelets per microliter of blood), heparin was not given. In left-sided vascular access, the left forearm was moved across the patient’s body so that the left palm would face the patient’s right groin. TACE was then carried out by the same experienced interventional radiologists (31 and 15 years of experience, respectively). 

A 5-Fr 125 cm hydrophilic–angiographic catheter (Cobra, Simmons, Multipurpose; Radifocus Glidecath; Terumo Corp, Tokyo, Japan) with a hydrophilic guide wire (Radifocus™ Guide Wire M Standard Type 0.035” 260 Cm Angled; Terumo Corp, Tokyo, Japan) was used for the selective celiac trunk catheterization and the cannulation of the common hepatic artery. Subsequently, the remaining endovascular procedural steps were performed as previously described [19].Whenever possible, a superselective approach (via tumor-feeding vessels) was obtained using the above microcatheter; alternatively, a pattern of segmental, lobar, or whole-liver (proper hepatic artery) chemoembolization was followed. LifePearl microspheres 200 ± 50 µm (Terumo Corp, Tokyo, Japan) were loaded with Doxorubicin, at a dose of 75 mg/m^2^ body surface area, and non-ionic iodinated contrast media, according to the manufacturer’s instructions for use. The mixture of doxorubicin-loaded microspheres and non-ionic iodinated contrast media was injected slowly. If blood stasis had not been achieved at the end of the target chemotherapy dose, a small additional dose of microspheres was used. If the target vessel was filled with contrast without washout for at least 5 heartbeats, stasis was achieved [20]. Angiographies of the contralateral lobar branch of the hepatic artery, superior mesenteric artery, and abdominal aorta were performed to identify any residual tumor-feeder arteries. After the procedure was completed, the sheath was flushed and pulled out. Early hemostasis was achieved by manual compression; subsequently, a radial artery compression device (TR Band^®^; Terumo Corp, Tokyo, Japan) was applied at the snuffbox (operators were instructed to use the minimum volume of air to maintain hemostasis). Patent hemostasis was the goal of preventing radial artery occlusion [21]. The hemostatic device-removal time was then recorded. Assessment of vascular access-site complications (VASCs), including radial artery occlusion, was performed at the patient’s discharge and at 4 weeks after each treatment by clinical assessment and doppler ultrasound. Patient follow-up and possible repetition of the treatment followed the same schedule and indications as previously described [19].

### 2.3. Outcomes and Definitions

The primary efficacy endpoint was the technical success rate achieved by distal radial access. Secondary efficacy endpoints included the conversion rate and the rate of successful cannulation and sheath introduction. The primary safety endpoint was the rate of VASCs. Adverse events and clinical complications linked to chemoembolization were selected as secondary safety endpoints.

Distal transradial access was the vascular access performed at the distal part of the radial artery, located at the anatomical snuffbox as described by Kiemeneij [6]. Vascular access-site conversion, summarized as “conversion rate”, was the crossover to another vascular access site. Success cannulation and sheath introduction was defined by successful wiring and positioning of the introducer sheath and included those patients requiring vascular access-site conversion due to technical difficulties in completing TACE. Radial artery spasm (RAS) and radial artery occlusion (RAO) were diagnosed by angiography and doppler ultrasound, respectively. Major bleeding was defined by a decrease in blood hemoglobin concentration greater than 3 g/dL. Cannulation time was defined as the time between the end of local anesthesia administration and the flushing of the introducer sheath immediately after its positioning. The absence of bleeding and hematoma after release defined the success of hemostasis. The number of punctures was calculated considering each time the needle was pulled out of the skin after its insertion. Technical success was defined as the ability to deliver the full planned dose of Doxorubicin and to obtain stop flow [22]. Treatment response was assessed using mRECIST guidelines [23]. Adverse events linked to chemoembolization were graded using the National Cancer Institute Common Terminology Criteria for adverse events (CTCAE) version 5.0 (National Cancer Institute, 2017), except for clinical complications associated with chemoembolization recorded using the CIRSE Classification System for Complications [24].

### 2.4. Statistical Analysis

Data were maintained in an Excel spreadsheet (Microsoft Inc, Redmond, Wash), and the statistical analyses were performed on an intention-to-treat basis, using SPSS software (SPSS, version 22 for Windows; SPSS Inc., Chicago, IL, USA). A Kolmogorov–Smirnov test and Shapiro–Wilk test were used to verify the normality assumption of data. Categorical data are presented as frequency (percentage value). Continuous normally distributed data are presented as mean ± standard deviation. Continuous, not normally distributed data are presented as median (interquartile range: 25th and 75th percentiles—IQR). The unpaired Student t-test was used to assess statistical differences for continuous normally distributed data, while categorical and continuous not normally distributed data were assessed using the chi-squared/Fisher’s exact tests and the Mann–Whitney test, respectively. A *p*-value of <0.05 was considered statistically significant for the aforementioned tests.

## 3. Results

### 3.1. Study Population

During the study interval (January 2018–December 2022), 42 patients underwent transcatheter arterial chemoembolization of the liver with distal radial access. Three patients were excluded from the preprocedural evaluation because the distal radial artery diameter was not suitable for distal transradial access (dTRA) according to exclusion criteria. 

The mean age was 53.1 years, and 66.7% of the patients were male. The mean value of the body mass index was 25 (±5.3). The median alpha-fetoprotein and carbohydrate antigen 19-9 levels were 547 ng/mL and 4 U/mL, respectively. Thirty-eight patients (90.5%) were affected by cirrhosis; all patients were in Child–Pugh class B. The median (IQR) maximum tumor size was 5.1 cm (3.6–5.5 cm). 

Demographics, comorbidities, and liver disease data for the study population are reported in Table 1.

### 3.2. Procedure Data

**Figure 1:**
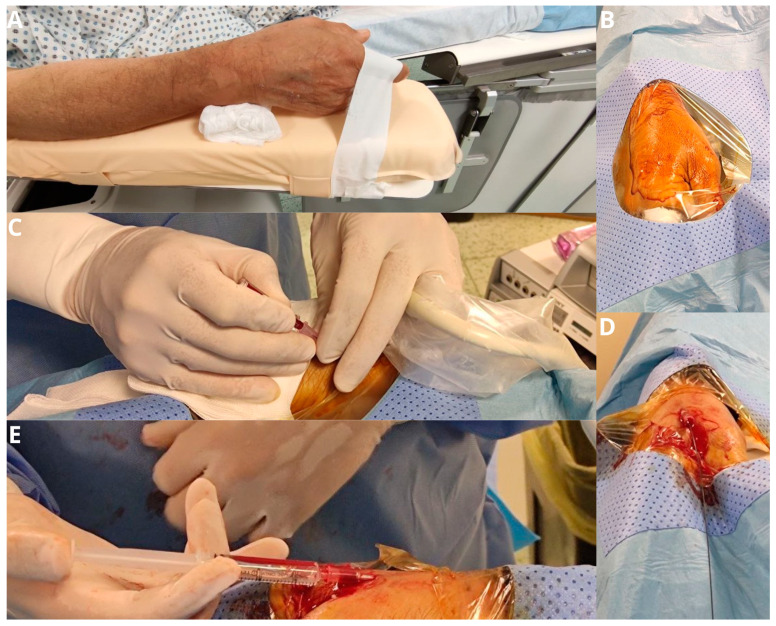
The patient’s forearm is positioned in a neutral semi-pronated position (**A**) and a drape is placed over the hand after it is prepped, exposing the anatomical snuff box (**B**). Arterial puncture of the distal radial artery is performed with a 21-gauge micro-puncture needle under ultrasound guidance (**C**). The wire is advanced through the needle in the vessel in a retrograde fashion (**D**). If there is any resistance in passing the wire further in the forearm, further imaging with fluoroscopy and angiography is performed (**E**).

**Figure 2:**
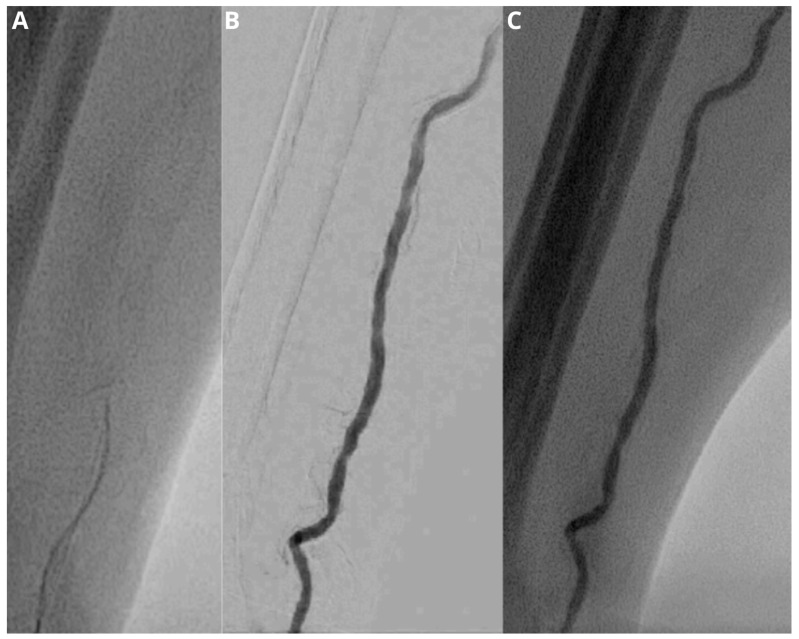
Fluoroscopic image (**A**) demonstrates a difficult advancement of the wire. Subsequent injection of contrast medium under fluoroscopy and digital subtraction angiography (**B**,**C**) shows a vessel tortuosity, which makes successful cannulation and sheath introduction not possible.

Forty-two TACE of the liver were performed; in all cases, technical success was achieved. The mean vessel size of the distal radial artery was 2.52 (±0.25) mm, with a mean number of vascular access-site punctures per patient of 1.4 (±0.5). An average cannulation time of 273.2 (±53) seconds was recorded. The conversion rate was 2.4%, related to a case of unsuccessful cannulation and sheath introduction due to vessel tortuosity (Figure 1 and Figure 2). The mean time to hemostasis was 13.1 (±1.5) minutes. The mean procedure duration was 68.3 (±9.9) minutes, with a mean fluoroscopy time of 26.8 (±6.2) minutes. Radiation exposure expressed by total air kerma and the total dose-area product was recorded as 509 (213–1518) mGy and 76 (44–198) Gy/cm^2^, respectively. 

Procedure data are detailed in Table 2.

### 3.3. Safety Outcomes

The rate of vascular access-site complications (VASCs) was 4.8%, related to two cases of hematoma. No episode of radial artery spasm, radial artery occlusion, arteriovenous fistula, pseudoaneurysm, retrograde dissection, or major bleeding occurred.

According to the CIRSE classification system for complications, 14 patients (33.3%) experienced postprocedural clinical complications associated with chemoembolization. Apart from four (9.5%) treatment-related grade 3 events (2 non-surgical cholecystitis and 2 prolonged compressions of the vascular access site), only grade 1 events occurred (10 cases, 23.8%). These were pain relieved by analgesics (4 cases, 9.5%), post-embolization syndrome (2 cases, 4.8%), transient nausea (2 cases, 4.8%), and vomiting (2 cases, 4.8%). The aforementioned adverse events were transient and easily solved with standard analgesic or antiemetic medication during interventions. 

Details are given in Table 3.

### 3.4. Distal Radial Access vs. Femoral Access

During the same study interval (January 2018–December 2022), 40 patients underwent DEB-TACE with femoral access. No statistically significant differences were observed between the group undergoing DEB-TACE with distal radial access and the group undergoing DEB-TACE with femoral access in terms of age, gender, BMI, prothrombin time, alpha-fetoprotein, cirrhosis, Child–Pugh class, platelet count, number of tumors, maximum tumor size, technical success, conversion rate, chemoembolization patter, procedure duration, vascular access-site complications, and post-procedural clinical complications. The cannulation time was significantly higher in the DEB-TACE group with distal radial access (273.2 s vs. 196.6 s; *p* < 0.0001).

Details are given in Table 4.

## 4. Discussion

The distal radial artery (dRA) has recently emerged as an alternative vascular-access site to conventional transfemoral and transradial approaches in interventional cardiology and interventional radiology/neuroradiology procedures [6,9,25].

Since its first description by Kiemeneij [6], the distal radial artery approach has shown some advantages over the conventional radial approach, including the decreased risk of radial artery occlusion and hand ischemia, the increased possibility of radial artery reuse, the decreased time to hemostasis, the improved operator and patient comfort for left-sided approaches and the applicability to patients with various orthopedic injuries (frozen injuries, etc.) limiting the wrist supination.

Data regarding this approach in interventional radiology procedures are limited to a few case-series studies in the absence of data collected exclusively for chemoembolization of the liver [8,9,10].

The DAPRAO (Distal Radial Approach to Prevent Radial Artery Occlusion) trial highlighted a striking 91.5% relative risk reduction of radial artery occlusion (RAO) proximal to the radial styloid process, with distal radial access compared to conventional radial access at the wrist, yielding a number needed to treat of 13 [26]. The findings likely reflect that the point of vascular access-site puncture, sheath introduction, and subsequent compression for hemostasis is the area at the highest risk of thrombosis and occlusion [27]. The anatomy and physiology of upper limb vascularization provide important clues toward a better understanding of the aforementioned finding [28]. If thrombosis occurs at the conventional radial cannulation site just proximal to the styloid process of the radius bone, it can extend retrograde to the origin of the radial artery. In proximity to the occlusive site, there are no collateral circles capable of maintaining sufficient antegrade flow in the radial artery. Instead, if thrombosis occurs at the distal radial artery in the anatomic snuffbox, flow in the hand is maintained because the obstruction to flow is beyond the origin of the superficial palmar branch, thus preventing blood stasis during hemostasis and proximal thrombus growth [29,30]. The Distal Radial Access Doppler Study described an unchanged flow in the radial artery at the forearm during simulated RAO at the anatomic snuffbox level; on the contrary, a severe flow reduction was observed during simulated RAO at the wrist level [31]. Hence, distal radial access is associated with a reduced incidence of RAO, and this clinical finding is supported by a strong biological plausibility. Prevention of radial artery occlusion is of particular importance in those patients who, for various clinical reasons, have to undergo repeated endovascular procedures (TACEs, PCIs, etc.) or who should preserve the radial artery for possible subsequent coronary artery bypass grafting.

A dRA diameter equal to or greater than 2 mm was chosen to perform the vascular access site at the dRA. These values make the vessel/sheath diameters ratio > 1, considering the outer diameter (1.78 mm) of the sheath (Glidesheath Slender™, Terumo). This preprocedural assessment is critical to minimize the risk of arterial occlusion [32]. The ratio between the vascular diameters of the distal radial artery at the anatomical snuffbox and the radial artery at the wrist is about 0.8–0.9 [33]. Hence, successful cannulation of the distal radial artery is linked to anatomical factors that cannot be circumvented. The percentage of patients (7.1%) with distal radial artery diameters not suitable for distal transradial access (dTRA) is comparable with other published investigations in this field [34]. 

The mean number of attempts made to gain vascular access was slightly better than that reported by Aoi and Izumida [35,36], of 1.8 and 2.4, respectively. The constant use of ultrasound guidance could explain this finding. Ultrasound guidance is particularly useful when the pulse of the distal radial artery is weak.

Technical success was achieved by distal radial access in 41 (97.6%) cases; therefore, distal radial access proved to be highly effective in patients undergoing DEB-TACE. Du et al. reported a similar technical success rate (95% and 98.8%, respectively) in a cohort of 112 patients undergoing 160 TACEs via conventional radial access and in a cohort of 107 patients undergoing 163 TACEs via femoral access [37]. Park et al. observed a 97.9% technical success rate in 47 non-coronary endovascular procedures performed via distal radial access, including 19 (40.4%) TACE [38]. In our study, we did not observe significant differences in terms of efficacy endpoints between the group undergoing DEB-TACE with femoral access and the group undergoing DEB-TACE with distal radial access. Treatment response was in keeping with other investigations in the field [39], although this was not the focus of the study. Successful cannulation and sheath introduction were not possible only in one out of 42 patients (2.4%) due to vessel tortuosity despite fluoroscopic guidance. In their recent meta-analysis, Izumida and Liang [36,40] reported a dRA cannulation failure of 20.2% and 4.3%, respectively. The heterogeneity could be explained by patients discarded a priori from various investigations for anatomical factors, leading to potential selection bias. In our experience, three patients were excluded due to insufficient vessel diameter. If the vessel diameter were not chosen among the exclusion criteria, the cannulation failure would have risen up to 9.5%. The successful cannulation rate of the distal radial artery in patients undergoing TACE of the liver does not differ from those for coronary interventions. As stated by Liang [40], the overall cannulation failure of the distal radial artery is slightly higher than that of the radial artery at the wrist, without a statistically significant difference (4.3% vs. 3.8%, *p* > 0.05). 

In our case series, only a case of conversion to femoral access was recorded (2.4% conversion rate). In a recent case series of 112 patients who underwent 160 TACEs by conventional radial access [37], 1.9% of cases underwent crossover to femoral access for selective cannulation failure. Al-Azizi [41] reported a conversion rate of 1.7% due to a case of severe tortuosity of the left subclavian artery resulting in unsuccessful cannulation of the right coronary artery and an additional right radial artery cannulation. In patients undergoing TACE of the liver, the selective catheterization of the celiac trunk is generally favored by an antegrade approach, guaranteed by radial access, compared to the retrograde approach via femoral access. It is possible that in rare cases, severe tortuosity of the left subclavian artery obliges vascular access-site conversion, as it happened to Al-Azizi for coronary interventions.

Lee et al. [42] described a reduction in cannulation time until about 150 distal radial artery accesses are not performed; therefore, it is plausible that in our case series, the learning curve has not yet been overcome. However, Bhambhani [43] reported cannulation times similar to ours. In previous studies, definitions can vary from “cannulation time” to “access time” [35,44]. Beyond the terminological question, it is worth underlining that the lack of standardization of the moments defining the beginning and the end of the cannulation time may lead to a dangerous heterogeneity of the data, compromising the validity of future meta-analytic studies. However, cannulation time is significantly shorter for conventional radial access compared to distal radial access, according to Aoi et al. [35], at least until the learning curve is overcome. In our study, cannulation time was shorter for femoral access compared to distal radial access. If these data are not favorable for time-sensitive scenarios such as acute coronary syndromes or traumatic pelvic bleedings, this criticality loses its value in non-urgent endovascular procedures such as chemoembolization. Moreover, procedures like this and similar could be used deliberately during the initial phase of the learning curve before using the distal radial access in time-sensitive scenarios.

The distal radial access was performed on the right side in only 6 cases (14.3%) due to previous endovascular treatments (PCIs) in which the left radial artery was used as the vascular access site.

The total procedure duration of transcatheter arterial chemoembolization (TACE) of the liver with distal radial access does not differ from similar procedures with conventional vascular access sites [45]. Ishiguchi reported a similar mean fluoroscopy time in a case series of 40 patients undergoing chemoembolization of the liver for HCC with femoral access [46]. The dose-area product (DAP) does not differ from that of other investigations in this field in which a femoral access site was used [47].

For coronary angiography and intervention, Liang et al. found no significant difference in radiation DAP between distal and conventional radial access [40]. Access on the dorsal side of the hand allows the patient to hold the hand in a neutral semi-pronated position and, consequently, to place it on the right groin in a comfortable way. Besides, the operator is not forced to bend over on the patient’s left side approaching the radiation source nor to maneuver guides and catheters with his left hand if the patient’s left arm is placed slightly abducted from the body on an arm sling for the entire duration of the procedure.

The rate of vascular access site complications (VASCs) was comparable to other published studies in the endovascular field, whether it is femoral or radial access [35,40,41,44,48,49,50]. Two cases of access site hematoma in high-risk patients with a platelet count lower than 100,000 per microliter of blood were observed. In distal radial access, the bony base consisting of the scaphoid bone and trapezium bone promotes hemostasis so that major bleedings and hematomas are rare [51]. Koury described a rate of radial artery spasm (RAS) and radial artery occlusion (RAO) of about 2.7% and 0%, respectively, in a case series of 37 patients undergoing dRA access for abdominopelvic endovascular interventions [8]. Izumida and Liang reported RAS/RAO rates of about 1.6%/1.4% and 2.6%/1.7%, respectively [36,40]. In our experience, no episodes of radial artery spasm or radial artery occlusion have been recorded, probably favored by the small population under study. The evaluation of the radial artery’s patency was performed by Doppler ultrasound, contrary to other studies in which the evaluation of patency was performed only with clinical evaluation leading to a risk of false negatives. The low rate of RAO with dRA, evidenced by the data in the literature, could have various explanations: (1) ultrasound measurement of dRA diameter allows to exclude patients with unfavorable anatomy, leading to reduced endothelium damage [52]; (2) the shorter time to hemostasis [53]; (3) during distal radial artery compression, the risk of thrombus formation is minimized by the preserved antegrade flow through the superficial palmar arch [6].

According to both the CTCAE and the CIRSE classification systems, patients experienced a global rate of adverse events after chemoembolization that is comparable to previous data on cTACE, DSM-TACE, or DEB-TACE, performed with conventional femoral or radial accesses [19,20,39,54,55,56], and on other procedures in the endovascular fields [57,58,59]. Finally, in our investigation, we did not observe significant differences in terms of safety endpoints between the group undergoing DEB-TACE with femoral access and the group undergoing DEB-TACE with distal radial access.

The mean time to hemostasis was in keeping with those recorded in other studies for cardiac catheterization and percutaneous coronary interventions (PCIs) [35,60]. A recent meta-analysis by Izumida and Liang found that compared with cTRA, time to hemostasis and hemostatic device-removal time are shorter in dTRA [36,40]. There is a paucity of data in the literature regarding the volume of inflated air in the TR Band (Terumo^®^). Aoi et al. described a lower volume than that insufflated in the conventional radial access at the wrist [35]. The bone base closely attached to the distal radial artery could explain this finding.

Limitations related to the distal radial access should be noted: (1) anatomical factors limiting its use and the use of large bore sheaths; (2) the slower cannulation time leading to biological costs in time-sensitive scenarios, at least until the learning curve is overcome; (3) the need of ultrasound guidance to screen the suitable patients: a one-size-fits-all approach is contraindicated; (4) the effects on the scaphoid bone blood supply are unknown, considering that the scaphoid bone receives its blood supply primarily from lateral and distal branches of the radial artery [41].

Limitations of the study are the single-center setting, the small study population, the retrospectivity of the analysis, and the scarcity of data in the literature necessary to evaluate the congruence and consistency of the data presented.

## 5. Conclusions

To the best of our knowledge, no observational studies have so far investigated the efficacy and safety profile of distal radial access solely for transcatheter arterial chemoembolization (TACE) of the liver.

Hence, the results of the current investigation demonstrate that distal radial access is effective and safe in embolization procedures, achieving an interesting rate of vascular access-site complications (VASCs). Among embolization procedures, such data were observed in the population undergoing transcatheter arterial chemoembolization (TACE) of the liver, in which repeated endovascular chemoembolization was often performed so that prevention of radial artery occlusion is crucial. The safety and efficacy profile of the distal radial approach was comparable to that of the femoral approach in patients undergoing TACE for HCC treatment.

Larger, randomized, controlled trials are needed to confirm these preliminary data.

## Figures and Tables

**Table 1:** Population data.

Variables	All Patients (n = 42)
Age (years)	53.1 (±18.6)
Sex (M/F)	28 (66.7%)/14 (33.3%)
BMI	25 (±5.3)
Diabetes mellitus	20 (47.6%)
Coronary artery disease	22 (52.4%)
Congestive heart failure	18 (42.9%)
Cerebrovascular disease	8 (19.0%)
Smoking history	28 (66.7%)
Current smoker	18 (42.9%)
Hypertension	32 (76.2%)
Hyperlipidaemia	30 (71.4%)
Chronic renal insufficiency (eGFR <90 mL/min)	14 (33.3%)
Hepatitis B virus	6 (14.3%)
Hepatitis C virus	20 (47.6%)
Non-alcoholic fatty liver disease	2 (4.8%)
Alcoholic liver disease	19 (38.1%)
α-Fetoprotein (ng/mL)	547 (176–1878)
Carbohydrate antigen 19-9 (U/mL)	4 (0.4–40.9)
γ-Glutamyltransferase (U/L)	98 (8–989)
Alkaline phosphatase (U/L)	50 (22–861)
Aspartate transaminase (U/L)	34 (5–250)
Alanine transaminase (U/L)	45 (11–298)
Albumin (g/L)	28 (26–34)
Total bilirubin (mg/dL)	1.1 (0.4–1.8)
Prothrombin time (seconds prolonged)	6 (5–9)
Ascites	0 (0%)
Encephalopathy	10 (23.8%)
Cirrhosis	38 (90.5%)
Child–Pugh Class, A/B/C	0 (0%)/42 (100%)/0 (0%)
Platelet count (No. ×10^3^/μL)	99 (90–410)
Creatinine (mg/dL)	1.2 (0.5–1.5)
Hemoglobin (g/dL)	13.1 (11–14.9)
Number of tumors, 1/2/3	18 (42.8%)/12 (28.6%)/12 (28.6%)
Maximum tumor size (cm)	5.1 (3.6–5.5)
Bilobar disease	18 (42.8%)
Capsule	22 (52.4%)

**Table 2:** Procedure data.

Variables	
Total number of DEB-TACE	42
Technical success	42 (100.0%)
Technical success by distal radial access	41 (97.6%)
Vessel size (mm)	2.52 (±0.25)
Number of punctures of vascular access site	1.4 (±0.5)
Cannulation time (seconds)	273.2 (±53)
Side of vascular access, right/left	6 (14.3%)/36 (85.7%)
Successful cannulation and sheath introduction, no/yes	1 (2.4%)/41 (97.6%)
Vascular access site conversion	1 (2.4%)
Sheath diameter, 4F/5F/6F/7F	0 (0%)/42 (100%)/0 (0%)/0 (0%)
Intra-arterial unfractionated heparin	10 (23.8%)
Chemoembolization pattern - Superselective - Segmental - Lobar - Whole-liver	35 (83.3%) 5 (11.9%) 2 (4.8%) 0 (0%)
Time to hemostasis (min)	13.1 (±1.5)
TR band air inflation (mL)	10 (±0.3)
Hemostatic device-removal time (min)	128.8 (±26.6)
Contrast volume (mL)	77.9 (±12.5)
Procedure duration (min)	68.3 (±9.9)
Fluoroscopy time (min)	26.8 (±6.2)
Cumulative air kerma (mGy)	509 (213–1518)
Dose area product (DAP) (Gy/cm^2^)	76 (44–198)
Tumor response to DEB-TACE - Complete Response - Partial Response - Stable Disease - Progressive Disease	10 (23.8%) 12 (28.6%) 11 (26.2%) 9 (21.4%)

**Table 3:** Safety Outcomes.

Variables		
Vascular access-site complications (VASCs), no/yes		40 (95.2%)/2 (4.8%)
	Haematoma	2 (4.8%)
	Pseudoaneurysm	0 (0%)
	Retrograde dissection	0 (0%)
	AV Fistula	0 (0%)
	Major bleeding	0 (0%)
	Radial artery occlusion	0 (0%)
	Radial artery spasm	0 (0%)
Post-procedural clinical complications (CIRSE class.), absent/present		28 (66.7%)/14 (33.3%)
	Grade 1	10 (23.8%)
	Grade 2	0 (0%)
	Grade 3	4 (9.5%)
Adverse Events (CTCAE), absent/present		24 (57.1%)/18 (42.9%)
	Grade 1	8 (19.1%)
	Grade 2	8 (19.1%)
	Grade 3	2 (4.8%)
	Grade 4	0 (0%)

**Table 4:** Comparison of data between patients undergoing DEB-TACE with distal radial access and femoral access.

Variables	Group 1 (n = 42) Distal Radial Access	Group 2 (n = 40) Femoral Access	*p* Value
Age (years)	53.1 (±18.6)	57.9 (±15.9)	0.2346
Sex (M/F)	28 (66.7%)/14 (33.3%)	23 (57.5%)/17 (42.5%)	0.4953
BMI	25 (±5.3)	25.1 (±5.2)	0.8122
α-Fetoprotein (ng/mL)	547 (176–1878)	609 (234–1536)	0.8708
Prothrombin time (seconds prolonged)	6 (5–9)	8 (5–9)	0.1976
Cirrhosis	38 (90.5%)	37 (92.5%)	1
Child–Pugh class, A/B/C	0 (0%)/42 (100%)/0 (0%)	0 (0%)/40 (100%)/0 (0%)	1
Platelet count (No. × 10^3^/μL)	99 (90–410)	99 (98–109.8)	0.5061
Number of tumors, 1/2/3	18 (42.8%)/12 (28.6%)/12 (28.6%)	21 (52.5%)/10 (25%)/9 (22.5%)	0.7159
Maximum tumor size (cm)	5.1 (3.6–5.5)	5.2 (3.6–5.45)	0.7978
Technical success	42 (100.0%)	40 (100.0%)	1
Cannulation time (seconds)	273.2 (±53)	196.6 (±31.5)	<0.0001
Vascular access-site conversion	1 (2.4%)	0 (0%)	1
Chemoembolization pattern - Superselective - Segmental - Lobar - Whole-liver	35 (83.3%) 5 (11.9%) 2 (4.8%) 0 (0%)	34 (85%) 4 (10%) 2 (5%) 0 (0%)	1
Procedure duration (min)	68.3 (±9.9)	70 (±11.6)	0.5926
Vascular access-site complications (VASCs), no/yes	40 (95.2%)/2 (4.8%)	37 (92.5%)/3 (7.5%)	0.6718
Post-procedural clinical complications (CIRSE class.), absent/present - Grade 1 - Grade 2 - Grade 3	28 (66.7%)/14 (33.3%) 10 (23.8%) 0 (0%) 4 (9.5%)	21 (52.5%)/19 (47.5%) 14 (35%) 0 (0%) 5 (12.5%)	0.2604

## Data Availability

The data presented in this study are available on request from the corresponding author. The data are not publicly available due to privacy issues.

## Abbreviations

cTRA: conventional transradial access; DAP: dose-area product; dRA: distal radial artery; dTRA: distal transradial access; HCC: hepatocellular carcinoma; PCI: percutaneous coronary intervention; RAO: radial artery occlusion; RAS: radial artery spasm; TACE: transcatheter arterial chemoembolization; TRA: transradial access; US: ultrasound; VASCs: vascular access-site complications.

## References

[1] Posham R., Biederman D.M., Patel R.S., Kim E., Tabori N.E., Nowakowski F.S., Lookstein R.A., Fischman A.M. (2016). Transradial Approach for Noncoronary Interventions: A Single-Center Review of Safety and Feasibility in the First 1500 Cases. J. Vasc. Interv. Radiol..

[2] Ferrante G., Rao S.V., Jüni P., Da Costa B.R., Reimers B., Condorelli G., Anzuini A., Jolly S.S., Bertrand O.F., Krucoff M.W. (2016). Radial Versus Femoral Access for Coronary Interventions Across the Entire Spectrum of Patients With Coronary Artery Disease: A Meta-Analysis of Randomized Trials. JACC Cardiovasc. Interv..

[3] Kiemeneij F., Laarman G.J., Odekerken D., Slagboom T., van der Wieken R. (1997). A Randomized Comparison of Percutaneous Transluminal Coronary Angioplasty by the Radial, Brachial and Femoral Approaches: The Access Study. J. Am. Coll. Cardiol..

[4] Brueck M., Bandorski D., Kramer W., Wieczorek M., Höltgen R., Tillmanns H. (2009). A Randomized Comparison of Transradial versus Transfemoral Approach for Coronary Angiography and Angioplasty. JACC Cardiovasc. Interv..

[5] Valgimigli M., Gagnor A., Calabró P., Frigoli E., Leonardi S., Zaro T., Rubartelli P., Briguori C., Andò G., Repetto A. (2015). Radial versus Femoral Access in Patients with Acute Coronary Syndromes Undergoing Invasive Management: A Randomised Multicentre Trial. Lancet.

[6] Kiemeneij F. (2017). Left Distal Transradial Access in the Anatomical Snuffbox for Coronary Angiography (LdTRA) and Interventions (LdTRI). EuroIntervention.

[7] Hamandi M., Saad M., Hasan R., Megaly M., Abbott J.D., Dib C., Szerlip M., Potluri S., Lotfi A., Kiemeneij F. (2020). Distal Versus Conventional Transradial Artery Access for Coronary Angiography and Intervention: A Meta-Analysis. Cardiovasc. Revasc. Med..

[8] Koury A., Monsignore L.M., de Castro-Afonso L.H., Abud D.G. (2020). Safety of Ultrasound-Guided Distal Radial Artery Access for Abdominopelvic Transarterial Interventions: A Prospective Study. Diagn. Interv. Radiol..

[9] van Dam L., Geeraedts T., Bijdevaate D., van Doormaal P.J., The A., Moelker A. (2019). Distal Radial Artery Access for Noncoronary Endovascular Treatment Is a Safe and Feasible Technique. J. Vasc. Interv. Radiol..

[10] Shinozaki N., Ikari Y. (2022). Distal Radial Artery Approach for Endovascular Therapy. Cardiovasc. Interv. Ther..

[11] Llovet J.M., Brú C., Bruix J. (1999). Prognosis of Hepatocellular Carcinoma: The BCLC Staging Classification. Semin. Liver Dis..

[12] Forner A., Reig M.E., de Lope C.R., Bruix J. (2010). Current Strategy for Staging and Treatment: The BCLC Update and Future Prospects. Semin. Liver Dis..

[13] Reig M., Forner A., Rimola J., Ferrer-Fàbrega J., Burrel M., Garcia-Criado Á., Kelley R.K., Galle P.R., Mazzaferro V., Salem R. (2022). BCLC Strategy for Prognosis Prediction and Treatment Recommendation: The 2022 Update. J. Hepatol..

[14] European Association for the Study of the Liver (2018). EASL Clinical Practice Guidelines: Management of Hepatocellular Carcinoma. J. Hepatol..

[15] Oken M.M., Creech R.H., Tormey D.C., Horton J., Davis T.E., McFadden E.T., Carbone P.P. (1982). Toxicity and Response Criteria of the Eastern Cooperative Oncology Group. Am. J. Clin. Oncol..

[16] Barbeau G.R., Arsenault F., Dugas L., Simard S., Larivière M.M. (2004). Evaluation of the Ulnopalmar Arterial Arches with Pulse Oximetry and Plethysmography: Comparison with the Allen’s Test in 1010 Patients. Am. Heart J..

[17] Minici R., Siciliano M.A., Ammendola M., Santoro R.C., Barbieri V., Ranieri G., Laganà D. (2022). Prognostic Role of Neutrophil-to-Lymphocyte Ratio (NLR), Lymphocyte-to-Monocyte Ratio (LMR), Platelet-to-Lymphocyte Ratio (PLR) and Lymphocyte-to-C Reactive Protein Ratio (LCR) in Patients with Hepatocellular Carcinoma (HCC) Undergoing Chemoembolizations (TACE) of the Liver: The Unexplored Corner Linking Tumor Microenvironment, Biomarkers and Interventional Radiology. Cancers.

[18] Al-Azizi K.M., Lotfi A.S. (2018). The Distal Left Radial Artery Access for Coronary Angiography and Intervention: A New Era. Cardiovasc. Revasc. Med..

[19] Minici R., Ammendola M., Manti F., Siciliano M.A., Minici M., Komaei I., Currò G., Laganà D. (2021). Safety and Efficacy of Degradable Starch Microspheres Transcatheter Arterial Chemoembolization (DSM-TACE) in the Downstaging of Intermediate-Stage Hepatocellular Carcinoma (HCC) in Patients With a Child-Pugh Score of 8–9. Front. Pharmacol..

[20] Brown K.T., Do R.K., Gonen M., Covey A.M., Getrajdman G.I., Sofocleous C.T., Jarnagin W.R., D’Angelica M.I., Allen P.J., Erinjeri J.P. (2016). Randomized Trial of Hepatic Artery Embolization for Hepatocellular Carcinoma Using Doxorubicin-Eluting Microspheres Compared With Embolization With Microspheres Alone. J. Clin. Oncol..

[21] Edris A., Gordin J., Sallam T., Wachsner R., Meymandi S., Traina M. (2015). Facilitated Patent Haemostasis after Transradial Catheterisation to Reduce Radial Artery Occlusion. EuroIntervention.

[22] Basile A., Carrafiello G., Ierardi A.M., Tsetis D., Brountzos E. (2012). Quality-Improvement Guidelines for Hepatic Transarterial Chemoembolization. Cardiovasc. Intervent. Radiol..

[23] Lencioni R., Llovet J.M. (2010). Modified RECIST (MRECIST) Assessment for Hepatocellular Carcinoma. Semin. Liver Dis..

[24] Filippiadis D.K., Binkert C., Pellerin O., Hoffmann R.T., Krajina A., Pereira P.L. (2017). Cirse Quality Assurance Document and Standards for Classification of Complications: The Cirse Classification System. Cardiovasc. Intervent. Radiol..

[25] Kühn A.L., Singh J., Moholkar V.M., Satti S.R., Rodrigues K.d.M., Massari F., Gounis M.J., McGowan A., Puri A.S. (2021). Distal Radial Artery (Snuffbox) Access for Carotid Artery Stenting—Technical Pearls and Procedural Set-Up. Interv. Neuroradiol..

[26] Eid-Lidt G., Rivera Rodríguez A., Jimenez Castellanos J., Farjat Pasos J.I., Estrada López K.E., Gaspar J. (2021). Distal Radial Artery Approach to Prevent Radial Artery Occlusion Trial. JACC Cardiovasc. Interv..

[27] Hamilton G.W., Farouque O. (2021). Could Distal Radial Artery Access Do More Than “Just” Reduce Rates of Radial Artery Occlusion?. JACC Cardiovasc. Interv..

[28] Sgueglia G.A., Di Giorgio A., Gaspardone A., Babunashvili A. (2018). Anatomic Basis and Physiological Rationale of Distal Radial Artery Access for Percutaneous Coronary and Endovascular Procedures. JACC Cardiovasc. Interv..

[29] Sgueglia G.A., Santoliquido A., Gaspardone A., Di Giorgio A. (2021). Radial Artery Occlusion With Distal Radial Access Compared to Conventional Transradial Access: A Pathophysiology Outlook. JACC Cardiovasc. Interv..

[30] Richter Y., Edelman E.R. (2006). Cardiology Is Flow. Circulation.

[31] Sgueglia G.A., Santoliquido A., Gaspardone A., Di Giorgio A. (2021). First Results of the Distal Radial Access Doppler Study. JACC Cardiovasc. Imaging.

[32] Naito T., Sawaoka T., Sasaki K., Iida K., Sakuraba S., Yokohama K., Sato H., Soma M., Okamura E., Harada T. (2019). Evaluation of the Diameter of the Distal Radial Artery at the Anatomical Snuff Box Using Ultrasound in Japanese Patients. Cardiovasc. Interv. Ther..

[33] Yoshimachi F., Ikari Y. (2021). Distal Radial Approach: A Review on Achieving a High Success Rate. Cardiovasc. Interv. Ther..

[34] Meo D., Falsaperla D., Modica A., Calcagno M.C., Libra F., Desiderio C., Palmucci S., Foti P.V., Musumeci A.G., Basile A. (2021). Proximal and Distal Radial Artery Approaches for Endovascular Percutaneous Procedures: Anatomical Suitability by Ultrasound Evaluation. Radiol. Med..

[35] Aoi S., Htun W.W., Freeo S., Lee S., Kyaw H., Alfaro V., Coppola J., Pancholy S., Kwan T. (2019). Distal Transradial Artery Access in the Anatomical Snuffbox for Coronary Angiography as an Alternative Access Site for Faster Hemostasis. Catheter. Cardiovasc. Interv..

[36] Izumida T., Watanabe J., Yoshida R., Kotani K. (2021). Efficacy and Safety of Distal Radial Approach for Cardiac Catheterization: A Systematic Review and Meta-Analysis. World J. Cardiol..

[37] Du N., Yang M.-J., Ma J.-Q., Luo J.-J., Zhang Z.-H., Yu T.-Z., Zheng Z.-Y., Zhang W., Yan Z.-P. (2019). Transradial Access Chemoembolization for Hepatocellular Carcinoma in Comparation with Transfemoral Access. Transl. Cancer Res..

[38] Park S.E., Cho S.B., Baek H.J., Moon J.I., Ryu K.H., Ha J.Y., Lee S., Won J., Ahn J.-H., Kim R. (2020). Clinical Experience with Distal Transradial Access for Endovascular Treatment of Various Noncoronary Interventions in a Multicenter Study. PLoS ONE.

[39] Lammer J., Malagari K., Vogl T., Pilleul F., Denys A., Watkinson A., Pitton M., Sergent G., Pfammatter T., Terraz S. (2010). Prospective Randomized Study of Doxorubicin-Eluting-Bead Embolization in the Treatment of Hepatocellular Carcinoma: Results of the PRECISION V Study. Cardiovasc. Interv. Radiol..

[40] Liang C., Han Q., Jia Y., Fan C., Qin G. (2021). Distal Transradial Access in Anatomical Snuffbox for Coronary Angiography and Intervention: An Updated Meta-Analysis. J. Interv. Cardiol..

[41] Al-Azizi K.M., Grewal V., Gobeil K., Maqsood K., Haider A., Mohani A., Giugliano G., Lotfi A.S. (2019). The Left Distal Transradial Artery Access for Coronary Angiography and Intervention: A US Experience. Cardiovasc. Revasc. Med..

[42] Lee J.-W., Park S.W., Son J.-W., Ahn S.-G., Lee S.-H. (2018). Real-World Experience of the Left Distal Transradial Approach for Coronary Angiography and Percutaneous Coronary Intervention: A Prospective Observational Study (LeDRA). EuroIntervention.

[43] Bhambhani A., Pandey S., Nadamani A.N., Tyagi K. (2020). An Observational Comparison of Distal Radial and Traditional Radial Approaches for Coronary Angiography. J. Saudi Heart Assoc..

[44] Hammami R., Zouari F., Ben Abdessalem M.A., Sassi A., Ellouze T., Bahloul A., Mallek S., Triki F., Mahdhaoui A., Jeridi G. (2021). Distal Radial Approach versus Conventional Radial Approach: A Comparative Study of Feasibility and Safety. Libyan J. Med..

[45] Abdelsalam M.E., Figueira T.M.A., Ensor J., Tam A.L., Avritscher R., Kaseb A., Gupta S. (2022). The Impact of the Use of C-Arm Cone-Beam CT During Chemoembolization for Hepatocellular Carcinoma. Curr. Med. Imaging.

[46] Ishiguchi T., Nakamura H., Okazaki M., Sawada S., Takayasu Y., Hashimoto S., Hayashi N., Furui S., Koyama S., Maekoshi H. (2000). Radiation exposure to patient and radiologist during transcatheter arterial embolization for hepatocellular carcinoma. Nihon Igaku Hoshasen Gakkai Zasshi.

[47] Hidajat N., Wust P., Felix R., Schröder R.J. (2006). Radiation Exposure to Patient and Staff in Hepatic Chemoembolization: Risk Estimation of Cancer and Deterministic Effects. Cardiovasc. Intervent. Radiol..

[48] Minici R., Paone S., Talarico M., Zappia L., Abdalla K., Petullà M., Laganà D. (2020). Percutaneous Treatment of Vascular Access-Site Complications: A Ten Years’ Experience in Two Centres. CVIR Endovasc..

[49] Minici R., Ammendola M., Talarico M., Luposella M., Minici M., Ciranni S., Guzzardi G., Laganà D. (2021). Endovascular Recanalization of Chronic Total Occlusions of the Native Superficial Femoral Artery after Failed Femoropopliteal Bypass in Patients with Critical Limb Ischemia. CVIR Endovasc..

[50] Minici R., Serra R., Ierardi A.M., Petullà M., Bracale U.M., Carrafiello G., Laganà D. (2022). Thoracic endovascular repair for blunt traumatic thoracic aortic injury: Long-term results. Vascular.

[51] Feng H., Fang Z., Zhou S., Hu X. (2019). Left Distal Transradial Approach for Coronary Intervention: Insights from Early Clinical Experience and Future Directions. Cardiol. Res. Pract..

[52] Norimatsu K., Kusumoto T., Yoshimoto K., Tsukamoto M., Kuwano T., Nishikawa H., Matsumura T., Miura S.-I. (2019). Importance of Measurement of the Diameter of the Distal Radial Artery in a Distal Radial Approach from the Anatomical Snuffbox before Coronary Catheterization. Heart Vessels.

[53] Cai G., Huang H., Li F., Shi G., Yu X., Yu L. (2020). Distal Transradial Access: A Review of the Feasibility and Safety in Cardiovascular Angiography and Intervention. BMC Cardiovasc. Disord..

[54] Gruber-Rouh T., Schmitt C., Naguib N.N.N., Nour-Eldin N.A., Eichler K., Beeres M., Vogl T.J. (2018). Transarterial Chemoembolization (TACE) Using Mitomycin and Lipiodol with or without Degradable Starch Microspheres for Hepatocellular Carcinoma: Comparative Study. BMC Cancer.

[55] Lencioni R., de Baere T., Soulen M.C., Rilling W.S., Geschwind J.-F.H. (2016). Lipiodol Transarterial Chemoembolization for Hepatocellular Carcinoma: A Systematic Review of Efficacy and Safety Data. Hepatology.

[56] Minici R., Ammendola M., Manti F., Siciliano M.A., Giglio E., Minici M., Melina M., Currò G., Laganà D. (2021). Safety and Efficacy of Degradable Starch Microspheres Transcatheter Arterial Chemoembolization as a Bridging Therapy in Patients with Early Stage Hepatocellular Carcinoma and Child-Pugh Stage B Eligible for Liver Transplant. Front. Pharmacol..

[57] Minici R., Serra R., De Rosi N., Ciranni S., Talarico M., Petullà M., Guzzardi G., Fontana F., Laganà D. (2023). Endovascular treatment of femoro-popliteal occlusions with retrograde tibial access after failure of the antegrade approach. Catheter. Cardiovasc. Interv..

[58] Bracale U.M., Peluso A., Panagrosso M., Cecere F., DEL Guercio L., Minici R., Giannotta N., Ielapi N., Licastro N., Serraino G.F. (2022). Ankle-Brachial Index evaluation in totally percutaneous approach vs. femoral artery cutdown for endovascular aortic repair of abdominal aortic aneurysms. Chirurgia.

[59] Minici R., Venturini M., Fontana F., Guzzardi G., Pingitore A., Piacentino F., Serra R., Coppola A., Santoro R., Laganà D. (2023). Efficacy and Safety of Ethylene-Vinyl Alcohol (EVOH) Copolymer-Based Non-Adhesive Liquid Embolic Agents (NALEAs) in Transcatheter Arterial Embolization (TAE) of Acute Non-Neurovascular Bleeding: A Multicenter Retrospective Cohort Study. Medicina.

[60] Vefalı V., Sarıçam E. (2020). The Comparison of Traditional Radial Access and Novel Distal Radial Access for Cardiac Catheterization. Cardiovasc. Revasc. Med..

